# Supplementary material for: WHIRLY1 Acts Upstream of ABA-Related Reprogramming of Drought-Induced Gene Expression in Barley and Affects Stress-Related Histone Modifications
Source: Int J Mol Sci. 2023 Mar 28;24(7):6326. doi: 10.3390/ijms24076326 (PMC10094662; doi:10.3390/ijms24076326)
Supplement: Supplementary file 1 [file ijms-24-06326-s001.zip › Supplemental Figures and Tables legends.pdf]

Supplemental Figures and Tables legends

Figure S1:
Expression of *HvWHIRLY1* in primary leaves of wildtype and overexpression lines 2 and 15 at 28 das under drought (D) and control (C) conditions compared to wildtype control (which is set 1). Changes in relative expression level were measured via qPCR. Each graph bar represents the average value from 3 replications, the error bar shows the SEM. The character above the graph bar shows a statistically significantly different group from two-way ANOVA, Tukey test with $p<0.05$.

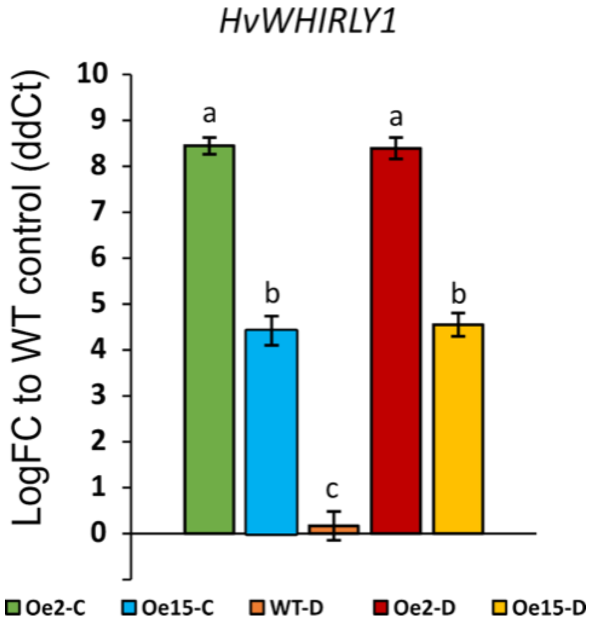

Table S1:
Primers used for qRT-PCR and ChIP-qPCR.

Table S2:
Excel files of differentially expressed genes. RNA sequencing was performed with primary leaves at 28 das from WT and oeW2, grown in control and drought conditions. Excel sheet “ALL DEGs” shows all differentially expressed genes (DEGs), with gene_id, expression levels under all conditions, gene description and further classification. In excel sheets “OEvsWT_C_up”, “OEvsWT-C_down”, “OEvs WT_D-up” and “OEvsWT_D-down” genes are listed according to their up- or down-regulation under control (C) and drought (D) conditions in overexpression line 2 compared to WT. In excel sheet “ABA synthesis related genes” the genes manually selected as ABA biosynthesis- related genes are listed.

Table S3:
Functional GO enrichment analysis of genes down- or up- regulated in overexpression line compared to wildtype. Functional classification to GO-terms (based on metabolic function (MF), biochemical process (BP) and cellular compartment (CC)), enrichment and q-values are shown. Description of GO-terms reveals that many of the down-regulated genes belong to functional casses “transport activities” (marked in yellow), “senescence processes” (marked in red), “hormone-related” processes (marked in magenta) and “biotic and abiotic stress-sensing” (marked in blue). Up-regulated genes have preferentially function in photosynthesis and chloroplast performance (marked in green).

Table S4:
Differentially expressed genes with WHIRLY-related cis-elements in promoter. In addition, functional annotation by GO-analysis of up- regulated genes with cis-elements in promoter are shown (photosynthesis and chloroplast-related functional classes are marked in green).
